# A medical illusion from Pinocchio

**DOI:** 10.1590/1516-3180.2014.1323748

**Published:** 2014-04-28

**Authors:** José Baddini-Martinez

**Affiliations:** I MD, PhD. Associate Professor, Division of Pneumology, Department of Internal Medicine, Faculdade de Medicina de Ribeirão Preto (FMRP), Universidade de São Paulo (USP), São Paulo, Brazil

Training doctors to be more humanistic has become a challenge for medical schools.^1^ ^-^ ^3^ Professionals with such training are able to establish better links with patients, thus leading to greater adherence to therapeutic interventions.^4^ This approach, besides promoting satisfaction and gratitude on the part of patients, also brings greater professional achievement and personal growth for physicians. Teaching humanistic behavior is a difficult task. Each moment of the medical course therefore must be seen as an opportunity for humanistic practice and learning. Some new methods of medical teaching have been developed over recent years.^5^ Actors are trained to simulate the symptoms of common medical conditions. Sophisticated mannequins make it possible to teach examination techniques, and enable training on invasive procedures for multidisciplinary teams.

Certainly, these resources are important for the technical preparation of health professionals, particularly for actions within critical scenarios. However, to what extent these new teaching methods contribute towards improvement of students' humanistic qualities is still unknown. Knowing the exact role of each member of the team responding to a cardiorespiratory arrest is extremely important, but learning how to deal with family members present at the scene is crucial as well. Learning the proper technique for examining an abdomen is indispensable, just as it is critical to know how to apply this technique to a patient who is in pain and fearful. Most probably, these aspects of physician-patient communication cannot be effectively developed without real human interactions.

Thus, we are now faced with an odd situation: the need to develop humanistic qualities in young medical students, alongside ease of access to artificial medical situations in controlled environments. As a consequence, a substantial number of professors and schools of Medicine seem to be experiencing excessive enthusiasm and unreasonable faith in simulation techniques, in what can be termed a medical illusion worthy of Pinocchio. It is worth remembering that Pinocchio was just a wooden puppet, yet with the extraordinary ability to speak and move, whose biggest dream was to become a real human being. Similarly, the excessive use of mannequins in medical education can speed up improvements in technical aspects of the profession but, most probably, does not contribute to the development of its humanistic aspects.

What, then, would be the best attitude for ensuring the learning of Medicine in the fullness of its aspects? Of course, invasive procedures should be practiced in simulation sessions, but medical students must be incorporated into service teams as soon as possible, and be given responsibility consistent with the degree of their practical experience. This traditional approach of teaching Medicine is laborious, tiresome and not without difficulties for both the teachers who are directly responsible for clinical teaching and for the patients. However, in this context, the simple act of asking for agreement permission to examine a sick person becomes a very special moment of human interaction. Medicine is truly learned only from patients or, to paraphrase Osler, it is advisable to say:

*"To teach the phenomena of disease without simulation is to sail an uncharted sea, while to teach them without patients is not to go to sea at all."* ^6^
